# Successful treatment of *Aspergillus* mural endocarditis in malignant lymphoma using a combined antifungal and surgical approach: a case report

**DOI:** 10.1186/s40792-020-00883-0

**Published:** 2020-06-05

**Authors:** Yuya Hiroshima, Soki Kurumisawa, Kei Aizawa, Noriyoshi Fukushima, Koji Kawahito

**Affiliations:** 1 Department of Cardiovascular Surgery, Jichi Medical University, Yakushiji 3311-1, Shimotsuke, Tochigi, 329-0498 Japan; 2 Department of Pathology, Jichi Medical University, Yakushiji 3311-1, Shimotsuke, Tochigi, 329-0498 Japan

**Keywords:** *Aspergillus* endocarditis, Fungal endocarditis, Malignant lymphoma, Immunosuppression, Case report

## Abstract

**Background:**

*Aspergillus* endocarditis (AE) is a rare and lethal cardiac infection with a high rate of mortality. AE most commonly presents in immunocompromised patients and is associated with various co-morbidities. Herein, we present a case of AE associated with lung, brain, and cervical abscesses after chemotherapy for malignant lymphoma that was successfully treated by a combination of antifungal and surgical therapy.

**Case presentation:**

A 29-year-old man was admitted to our hospital with an unidentified fever. He was diagnosed with malignant lymphoma (extra-nodal NK/T cell lymphoma nasal type), and chemotherapy was administered. After chemotherapy, nodular lung shadows along with new brain, cervical, and myocardial abscesses appeared, despite anti-bacterial/fungal therapy. Gene analysis of the cervical abscess biopsy revealed the presence of *Aspergillus fumigatus* species, and the transesophageal echocardiogram showed a mobile mural vegetation in the left ventricle (22 × 8 mm). He underwent surgical resection of this mural vegetation. His postoperative course was uneventful. He remains healthy at 28 months after surgery with continued oral antifungal therapy.

**Conclusion:**

Although AE associated with immunosuppression is a fatal clinical presentation, combined treatment with surgical resection and antifungal therapy was effective.

## Background

Fungal endocarditis remains the most rare and serious form of infective endocarditis, accounting for only 1–2% of all cases [1] with a high mortality rate of about 50% [2, 3]. Aspergillus endocarditis (AE) is a particularly severe form of fungal endocarditis. *Aspergillus* species account for 20–30% of fungal endocarditis cases, and mortality rates may reach up to 80–90% even with treatment [4, 5]. Furthermore, the incidence of AE increases in immunosuppressed patients. We herein report a salvaged case of AE associated with lung, brain, and cervical abscesses after chemotherapy for malignant lymphoma.

## Case presentation

A 29-year-old man with a history of chronic sinusitis was admitted to our hospital for an unidentified fever. He was diagnosed with malignant lymphoma (extra-nodal NK/T cell lymphoma nasal type), and two cycles of a dexamethasone, methotrexate, ifosfamide, L-asparaginase, and etoposide regimen (SMILE regimen) were administered. After the first chemotherapy cycle, he suffered septic shock due to *Staphylococcus haemolyticus* and *Corynebacterium striatum* infections and progressed to multi-organ failure. Although he required temporary mechanical ventilation for respiratory support and hemodialysis, the anti-bacterial/fungal therapy (meropenem hydrate, vancomycin, sulfamethoxazole, trimethoprim, and micafungin) controlled his bacterial infection and he recovered from his septic status. However, his fever persisted and nodular lung shadows (on day 27) along with new brain (on day 49), cervical, and myocardial abscesses (on day 53) appeared on computed tomography (CT). He underwent an aspiration biopsy of the cervical abscess on the 56th hospital day. Gene analysis of the cervical abscess revealed the presence of *Aspergillus fumigatus*. Although liposomal amphotericin B and voriconazole were administered, the patient remained febrile and CT on day 64 revealed increased sizes of brain abscesses. After articulatory and consciousness disorders (somnolence) appeared, burr hole drainage of the brain abscesses was emergently performed on the 70th hospital day. *Aspergillus* was not isolated from the burr hole drainage fluid. His neurological disorders immediately resolved after surgery.

**Fig. 1:**
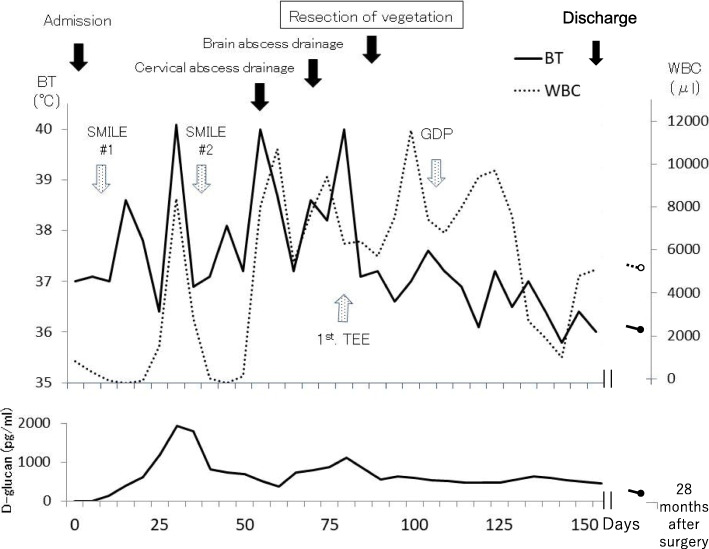
The perioperative clinical course. Before surgical resection of the vegetation, spiking fever, leukocytosis, and high D-glucan levels had persisted despite the anti-bacterial/fungal therapy. However, they were ameliorated after surgery. The leukocyte counts exhibited large fluctuations because of the hemophagocytic syndrome due to the malignant lymphoma and repeated chemotherapy (his biochemical presentation at the admission was pancytopenia due to hemophagocytic syndrome). BT, body temperature; WBC, white blood cell; TEE, transesophageal echocardiography; SMILE therapy, dexamethasone, methotrexate, ifosfamide, L-asparaginase, etoposide; GDP therapy, gemcitabine, dexamethasone, cisplatin

Despite the antifungal/bacterial therapy, his spiking fever remained and echocardiography performed on the 78th hospital day revealed mobile mural vegetation in the left ventricle (22 × 8 mm). Previous transthoracic echocardiography had failed to identify any mural vegetation. As this large mobile vegetation was thought to be the focus of his systemic fungal infection and a source of mycotic embolization to the vital organs, he was referred for surgery (Fig. 1).

**Fig. 2:**
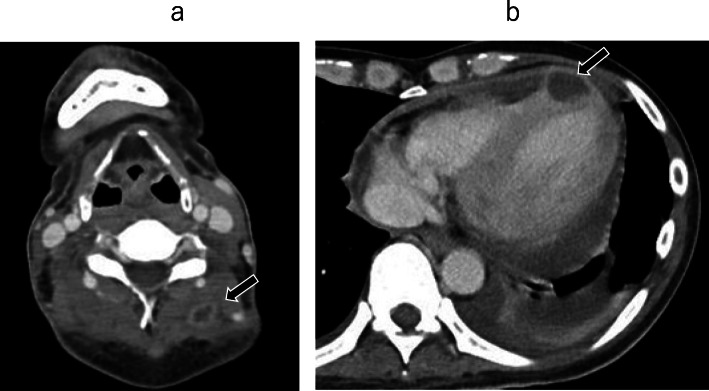
Preoperative contrast-enhanced computed tomography findings. Contrast-enhanced computed tomography revealed intramuscular low-enhancing lesions in the left posterior cervical region (**a**: arrow) and low-enhancing lesions in the left ventricle wall (**b**: arrow)

**Fig. 3:**
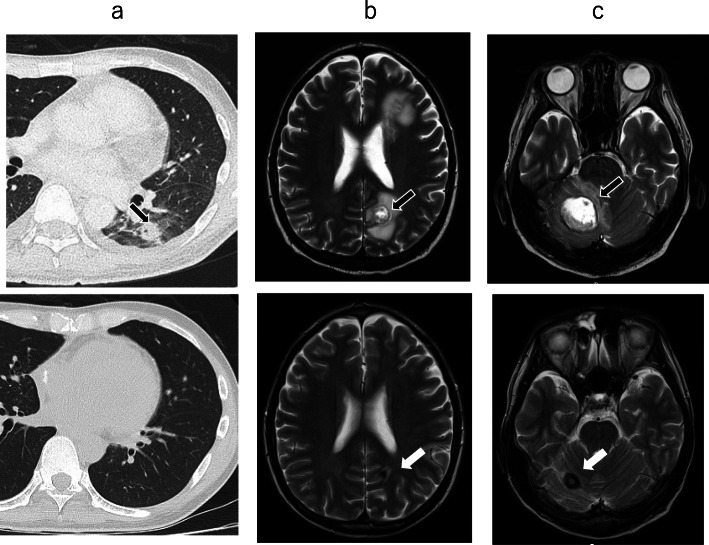
Pre- and postoperative chest computed tomography findings and brain magnetic resonance imaging findings. Preoperative contrast-enhanced chest computed tomography revealed nodular lung shadows with a small cavity in the left lower lobe (**a**—upper: arrow). Chest computed tomography performed at 3 months after surgery revealed no nodular lung shadows in the left lower lobe (**a**—lower). Magnetic resonance imaging demonstrated multiple bilateral rim-enhancing lesions with surrounding vasogenic edema in the left parietal lobe (**b**—upper: black arrow) and the cerebellum (**c**—upper: black arrow). The brain abscesses in the left parietal lobe (**b**—lower: white arrow) and cerebellum (**c**—lower: white arrow) had healed on the magnetic resonance imaging performed at 1 year after surgery

**Fig. 4:**
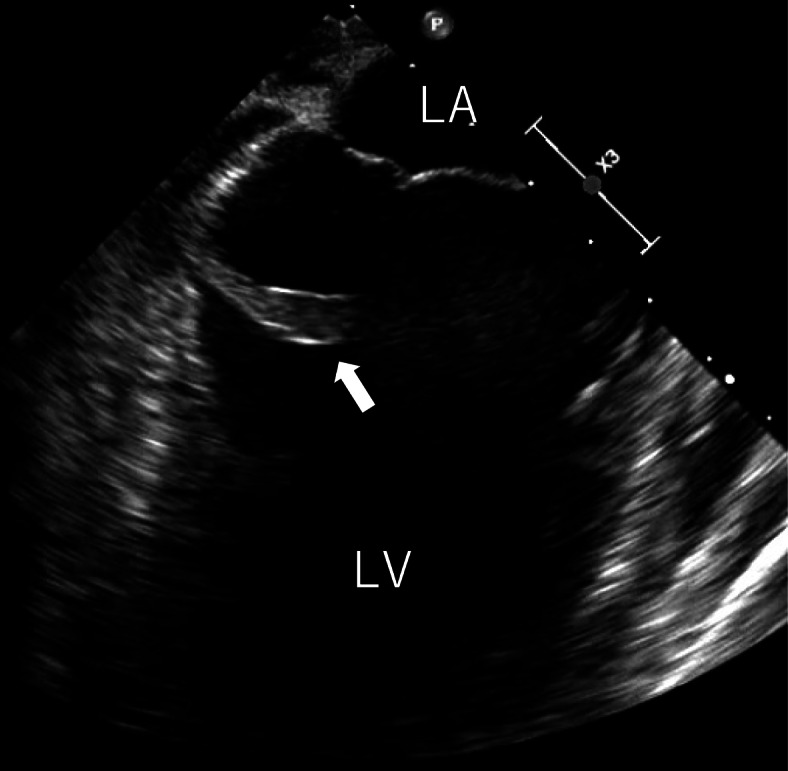
Preoperative transesophageal echocardiography findings. Transesophageal echocardiography revealed a mobile club-shaped mass of 22.0 × 8.0 mm on the basal posterior wall of the left ventricle. LA, left atrium; LV, left ventricle


On physical examination before surgery, his blood pressure was 113/91 mmHg, pulse 130 beats per minute and regular, and temperature 38.2 °C. Heart sounds were regular with no audible murmur present. He had no known history of treatment for chronic sinusitis, and especially no family history. Laboratory data showed a white blood cell count of 5.7 × 103/μL, a low hemoglobin level of 7.5 g/dL, thrombocytopenia with a platelet count of 98 × 103/μL, and increased C-reactive protein level at 4.72 mg/dL. Blood tests revealed an abnormally high serum β-D-glucan level (1120 pg/mL) and were positive for the *Aspergillus* antigen. A chest X-ray demonstrated consolidation in the left lower lobes. Contrast-enhanced CT showed multiple brain abscesses, an intramuscular abscess in the left posterior cervical region (Fig. 2a), intramuscular abscesses in the left ventricle (Fig. 2b), and left lung abscesses (Fig. 3a upper). Brain magnetic resonance imaging (MRI) revealed multiple bilateral rim-enhancing lesions with surrounding vasogenic edema (Fig. 3b, c upper). A transesophageal echocardiogram revealed a mobile mass measuring 22.0 × 8.0 mm in the left ventricle (Fig. 4, Suppl.1).

**Additional file 1.** Suppl.1 Transesophageal echocardiogram.


**Fig. 5:**
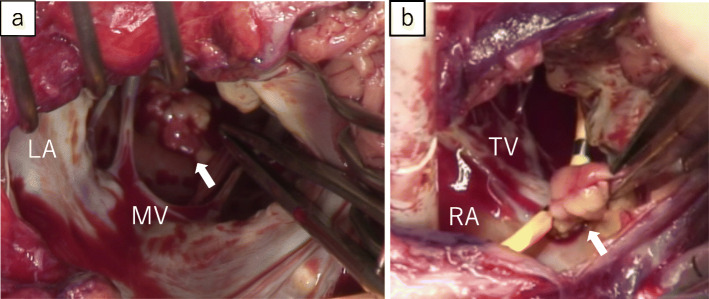
Intraoperative images. The intraoperative photograph shows mobile vegetation, 2.0 cm in diameter, attached between the papillary muscle and the posterior wall (**a**: arrow), and a small vegetation, 5 mm in diameter, adherent onto the septal and posterior commissure of the tricuspid valve (**b**: arrow). LA, left atrium; RA, right atrium; MV, mitral valve; TV, tricuspid valve

**Fig. 6:**
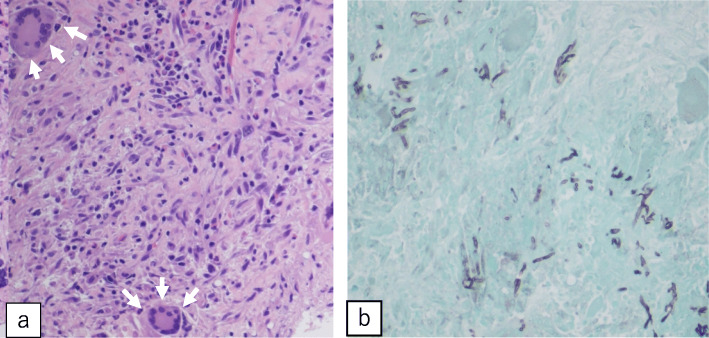
Histological findings. Photomicrograph of the left ventricular mass demonstrating cardiac muscle and necrotic debris with multinucleated giant cell invasion (**a**: arrows) (H&E stain, × 20 magnification) and *Aspergillus* species hyphae (**b**) (Grocott’s stain, × 20 magnification)

Elective surgery was performed on the 85th hospital day through a median sternotomy under cardioplegic arrest with standard cardiopulmonary bypass. We reached the mural vegetation on the left ventricle via a superior trans-septal approach. The mobile vegetation measuring 2.0 cm was positioned between the papillary muscle and the posterior wall and was resected. In addition to this vegetation, a small vegetation measuring 5 mm in diameter was observed on the septal and posterior commissure of the tricuspid valve. Although this vegetation had not been detected before surgery by echocardiogram, it was identified intraoperatively. As this vegetation extended towards the right ventricular wall, it was removed via wedge resection of the septal/posterior leaflet within the right ventricular wall (Fig. 5a, b). The defect in the tricuspid valve was repaired by 5-0 polypropylene. The patient’s postoperative course was uneventful. Pathology and cultures demonstrated the growth of *Aspergillus fumigatus* (Fig. 6a, b).

Anti-bacterial/fungal therapy with intravenous teicoplanin (600 mg/day), voriconazole (600 mg/day), and micafungin sodium (300 mg/day) was started after surgery, and his condition was stabilized. This regimen was continued for 12 weeks until discharge. During this hospitalization, he received additional chemotherapy (GDP therapy: gemcitabine, dexamethasone, cisplatin) and was discharged on postoperative day 84 after surgery (168th hospital day).

Chemotherapy was continued in the subsequent hospitalization, during which he was treated with high-dose chemotherapy and peripheral blood stem cell autotransplantation and achieved complete remission at 7 months after surgery. Contrast-enhanced chest CT performed at 3 months after surgery revealed no nodular lung shadows in the left lower lobe (Fig. 3a lower). The brain abscesses in the left parietal lobe and cerebellum had healed as demonstrated on MRI at 1 year after surgery (Fig. 3b, c lower). At 28 months after surgery, he remained healthy and continued with oral voriconazole (300 mg/day).

## Discussion

AE is an extremely rare infection, and mortality rates may reach 80–90% even with treatment [4–6]. Immunocompromised patients, those that have undergone prior cardiac surgery for cardiac abnormalities, and those with prosthetic heart valves have a particularly high risk for systemic fungal endocarditis. Other risk factors include parenteral nutrition, indwelling central venous catheters, prolonged use of broad-spectrum antibiotics, evolving myelodysplastic syndrome, immunosuppression caused by the use of steroid/cytotoxic drugs, and bone marrow transplantation with high-dose immunosuppressive therapy [7, 8]. In our case, immunosuppressed status due to malignant lymphoma and chemotherapy was the most obvious predisposing factors for AE.

A diagnosis of AE is usually difficult to reach because of the vague nature of its clinical presentation. Common clinical findings in patients with endocarditis include fever, changing heart murmurs, characteristically large peripheral emboli, and chorioretinitis [5]. Laboratory findings, including anemia, leukocytosis, erythrocyte sedimentation, and C-reactive protein, are not specific. In addition, positive blood cultures are obtained in a relatively small number of patients (sensitivity for blood cultures is only 4%) [9]. *Aspergillus* species almost never grow in blood cultures and, thus, must be isolated from removed emboli, diseased valves, or infected foreign bodies. Recent advances in serologic methods, including the use of *Aspergillus* antigens/anti-*Aspergillus* antibody, galactomannan antigens, and β-D-glucan, have enabled early detection; however, the problems of false-positives and low specificity remain. Meanwhile, Ellis et al. [6] reported that identification of vegetation by transthoracic and transesophageal echocardiography was effective for the diagnosis of fungal endocarditis, including AE. In AE, vegetations are often large and affect the mitral valve in 49% of cases, the aortic valve in 45%, the tricuspid valve in 17%, cardiac device leads in 9%, and the pulmonary valve in 2% [5]. Multi-valve involvement is observed in 21% of cases [5]. As for mural involvement of *Aspergillus*, it has been reported to be up to 40% in autopsy cases of AE [10]. Although transthoracic echocardiography failed to detect vegetation in this patient, transesophageal echocardiography, in addition to the serological findings and gene analysis of the cervical abscess, confirmed the diagnosis.

The neurological risks associated with cardiac surgery in patients with fungal brain abscesses and the optimal timing between the brain and cardiac operations have not been fully elucidated. In this case, bleeding was not observed and a subsequent CT showed the abscess to be encapsulated. Based on these findings, our heart team, which included neurologists, determined that the risk of systemic complications caused by mobile vegetation exceeded the risk of neurological complications. Theoretically, large mobile vegetations should be immediately removed; however, emergent surgery was considered to carry an excessive mortality risk in such a critically sick patient. Therefore, elective surgery was performed after obtaining informed consent.

The prognosis of patients with AE treated with medical therapy alone is poor, with an estimated mortality rate of 96% [5]. In general, a combined surgical-medical approach would yield the best results; however the survival rate after aggressive surgical debridement combined with antifungal treatment is not satisfactory at 68% [5]. Regarding antifungal therapy for invasive pulmonary *Aspergillosis*, voriconazole is recommended as the first-line treatment. Herbrecht et al. reported that voriconazole was associated with improved survival over amphotericin B, along with less nephrotoxicity, electrolyte abnormalities, and infusion-related events [11]. However, its effects as an antifungal therapy after surgical resection of intra-cardiac vegetation have not been well-documented. In this case, the persistent high fever and laboratory findings, including D-glucan level, improved after resection of the vegetation. Furthermore, the lung nodule that had appeared on chest CT disappeared at 3 months after surgery, and the brain abscess had healed on the MRI performed at 1 year after surgery. These findings suggested that the surgical resection helped treat the multiple organ aspergillosis along with the antifungal therapy. Our patient was administered voriconazole and micafungin sodium after surgery for 12 weeks, and oral voriconazole was continued after his discharge from hospital.

## Conclusion

We presented a rare case of *Aspergillus* mural endocarditis in an immunocompromised patient who survived. Combined antifungal therapy and surgical resection of the vegetation is essential to salvage such critically ill patients.

## Data Availability

Data sharing is not applicable to this article, as no datasets were generated or analyzed during the current study.

## Abbreviations

AE: *Aspergillus* endocarditis
CT: Computed tomography
MRI: Magnetic resonance imaging
